# The Pay of the Nurse. V.—A Comparison with Other Occupations
*Previous articles appeared March 5, 12, 26, and April 2.


**Published:** 1921-04-09

**Authors:** 


### THE MATRONS' AND SISTERS' DEPARTMENT.

### THE PAY OF THE NURSE.*

### V.—A Comparison with Other Occupations.

At the age of twenty-five or twenty-six the nurse is merely a staff-nurse, starting on the lowest rung of private nursing, not yet sufficiently experienced to secure a good post, possibly still undergoing training for admission to the Jubilee Institute, or taking midwifery training at her own expense as a prelude to an appointment requiring the C.M.B. certificate. She is lucky if, living in, she gets £1 a week. It is interesting to consider what her prospects would have been had she taken up some other branch of work.

Clerks and typists are a very large body, estimated to number some 300,000. Many of them pass straight from a board school education to a short course of training and at once get a weekly wage of 25s. to 35s. a week. After two or three years' experience, say at twenty or twenty-one, they are earning £2 5s. to £2 10s. a week. These are average clerks, often, it is to be feared, extraordinarily ignorant of all except their craft. It is believed by those who are attempting to organise this rather heterogeneous body of workers that their rate of pay is kept down owing to the fact that many of them live at home and have to pay merely a contribution towards the actual cost of their food and lodging. In other words, they are not obliged to have a living wage. When out of work, which frequently happens, they are found to prefer almost any privation to entering a living-in employment such as nursing. Better educated women with two or three years' experience can earn from £3 to £4 a week, and command of a foreign language or any other special acquirement would place £5 a week within the reach of the most efficient.

To turn to the profession of teaching, which most nearly compares with that of nursing. Teachers fall into two main classes: (1) in elementary, and (2) in secondary schools. The lowest scale for certificated women teachers, two years' college trained, girls of twenty or twenty-one, is £160, rising by annual increments of £10 to £272. Such as have three years of college training get an additional increment of £10, and four years' training commands an extra £20. Head-teacherships vary according to size of school, but command maxima varying from £300 to £412.

Passing to openings in secondary schools, the kind of work which women of higher education can obtain, and with which nursing will have to compete in the effort to secure High School and University women, we find the assistant mistress with university degree in a secondary school begins at the age of twenty-two or twenty-three at a minimum of £225, rising by annual increment of £15 to a maximum of £400, or if in London £275 and £440 respectively. She reaches the maximum in eleven years, and before the age of thirty-five is sure of an income which only a scanty group of matrons can ever hope to attain in the nursing world. Even without a degree she begins at a minimum of £177 10s., and rises by annual increment of £12 10s. to £320, or in London £197 and £360 respectively. There are also various allowances, including payments of an extra £40 a year for "posts of special responsibility," which make additions to the value of these appointments. Head-teacherships are numerous, and though no scale can be decided on owing to the varying types of school, the minimum commencing salary for Head Mistresses of secondary schools has been fixed at £500.

It is impossible to study these figures without being penetrated by a sense of the extent to which nurses have been and still are underpaid. It is a matter of importance to a far wider circle than those immediately affected. Nursing depends for its lifeblood on a perpetual stream of entrants. Without candidates the entire body of institutions for the sick, municipal, State, voluntary, becomes inoperative. Nurses as a class are devotedly loyal to their profession, and shoulder their burdens uncomplainingly. But it is the *potential* nurses who are striking. They dread the ill-assured future which stretches before them when once they enter the ranks. And without the concurrence of the candidates nursing cannot be carried on.

### ROUND THE HOSPITALS.

* Previous articles appeared March 5, 12, 26, and April 2.

